# Lactonase-mediated inhibition of quorum sensing largely alters phenotypes, proteome, and antimicrobial activities in *Burkholderia thailandensis* E264

**DOI:** 10.3389/fcimb.2023.1190859

**Published:** 2023-06-02

**Authors:** Mélanie Gonzales, Laure Plener, Jean Armengaud, Nicholas Armstrong, Éric Chabrière, David Daudé

**Affiliations:** ^1^ Aix Marseille Université, IRD, APHM, MEPHI, IHU-Méditerranée Infection, Marseille, France; ^2^ Gene&GreenTK, Marseille, France; ^3^ Université Paris-Saclay, CEA, INRAE, Département Médicaments et Technologies pour la Santé (DMTS), SPI, Bagnols-sur-Cèze, France; ^4^ Assistance Publique Hôpitaux de Marseille, APHM, Marseille, France

**Keywords:** quorum sensing, *Burkholderia thailandensis*, quorum quenching, lactonase, Acyl-homoserine lactone

## Abstract

**Introduction:**

*Burkholderia thailandensis* is a study model for *Burkholderia pseudomallei*, a highly virulent pathogen, known to be the causative agent of melioidosis and a potential bioterrorism agent. These two bacteria use an (acyl-homoserine lactone) AHL-mediated quorum sensing (QS) system to regulate different behaviors including biofilm formation, secondary metabolite productions, and motility.

**Methods:**

Using an enzyme-based quorum quenching (QQ) strategy, with the lactonase *Sso*Pox having the best activity on *B. thailandensis* AHLs, we evaluated the importance of QS in *B. thailandensis* by combining proteomic and phenotypic analyses.

**Results:**

We demonstrated that QS disruption largely affects overall bacterial behavior including motility, proteolytic activity, and antimicrobial molecule production. We further showed that QQ treatment drastically decreases *B. thailandensis* bactericidal activity against two bacteria (*Chromobacterium violaceum* and *Staphylococcus aureus*), while a spectacular increase in antifungal activity was observed against fungi and yeast (*Aspergillus niger*, *Fusarium graminearum* and *Saccharomyces cerevisiae*).

**Discussion:**

This study provides evidence that QS is of prime interest when it comes to understanding the virulence of *Burkholderia* species and developing alternative treatments.

## 1 Introduction

Most bacteria use a molecular communication system, referred to as quorum sensing (QS). It relies on the production and detection of small molecules known as autoinducers. QS enables the adaptation of bacterial behaviors in a cell density-dependent manner and allows bacteria to act cooperatively and induce different physiological functions (Mion et al., 2019). Among the mechanisms activated by QS, bioluminescence was the first described in *Vibrio* sp. (Tobias et al., 2020). Other mechanisms were later described, such as biofilm formation, virulence factor production, and antibiotic production (Zhao et al., 2020). Many autoinducers have been identified, including oligopeptides which are mainly used by Gram-positive bacteria, acyl-homoserine lactones (AHLs) by Gram-negative bacteria, and the diester AI-2 which is used by both (Rémy et al., 2018; Tobias et al., 2020). A wide panel of AHLs has been reported. These molecules share a lactone ring but their lateral acyl chain varies in term of modifications and/or length (Billot et al., 2020). Depending on the bacterial species, one or several specific AHLs can be produced and recognized by the cells to trigger virulence and biofilm formation . Biofilm formation allows bacteria to settle and limits the effectiveness of antimicrobial treatments (Rémy et al., 2018; Bowler et al., 2020). To counteract AHL-mediated bacterial virulence, strategies to disrupt QS, referred to as *quorum* quenching (QQ), have been developed. Various approaches have been reported, such as the use of natural or synthetic QS inhibitors (Rémy et al., 2018), autoinducer sequesters (Grandclément et al., 2016; Rémy et al., 2018) and QQ enzymes (QQEs), which can inactivate autoinducers (Rémy et al., 2018). One major advantage of QQ is that this strategy interferes with QS to prevent noxious behaviors without killing bacteria, thus minimizing the appearance of resistance mechanisms (Rémy et al., 2018). QQEs are particularly promising in light of their capacity to act catalytically and extracellularly for efficient QS disruption (Billot et al., 2020).

Among the QQEs, the archaeal enzyme *Sso*Pox (Hiblot et al., 2013) has been particularly investigated over the past decade considering its lactonase activity against a large panel of AHLs (Hiblot et al., 2013) and its tremendous resistance to temperature, process and long-term storage (Del Vecchio et al., 2009; Rémy et al., 2016). Enzyme engineering approaches made it possible to generate several *Sso*Pox variants with enhanced properties (Billot et al., 2022). The QQ potential of these variants has been demonstrated against different bacterial strains, including human pathogens (such as *Pseudomonas aeruginosa*) and environmental strains (such as *Chromobacterium violaceum*). Significant phenotypic alterations upon *Sso*Pox treatment have been reported, including biofilm disruption (Rémy et al., 2020; Mion et al., 2021), reduced virulence in *in vitro* (Rémy et al., 2020; Mion et al., 2021) and *in vivo* models (Hraiech et al., 2014), as well as modulation of antimicrobial production (Mion et al., 2021).

Here, the QQ effect of *Sso*Pox against *Burkholderia thailandensis* was evaluated. Besides some rare cases of human infections (Chang et al., 2017; Gee et al., 2018), *B. thailandensis* is of special interest as it constitutes a relevant study model for *Burkholderia pseudomallei* due to their high genome relatedness (Majerczyk et al., 2014b). *B. pseudomallei* is the causative agent of melioidosis (Klaus et al., 2018) and a potential bioterrorism agent (Oliveira et al., 2020). *B. thailandensis* constitutes a safe surrogate for studying and tackling *B. pseudomallei* virulence. Three QS systems were identified in *B. thailandensis*, each system being composed of an AHL synthase and a receptor to produce and sense a specific AHL signal. These three systems are shared with *B. pseudomallei* (Majerczyk et al., 2014b), QS-1 (BtaI1-BtaR1) QS-2 (BtaI2-BtaR2) and QS-3 (BtaI3-BtaR3) related to the production of C_8_-HSL, 3-OH-C_10_-HSL and 3-OH-C_8_-HSL respectively (Majerczyk et al., 2014b). In addition, two orphan receptors were also identified (Majerczyk et al., 2014a) (BtaR4 or MalR (Gupta et al., 2017) and BtaR5). QS regulates various phenotypes in *B. thailandensis* such as biofilm formation (Tseng et al., 2016), motility (Ulrich et al., 2004) and antimicrobial molecules including bactobolin (Klaus, 2020), 4-hydroxy-3-methyl-2-alkenylquinoline (Majerczyk et al., 2014a; Majerczyk et al., 2014b) and malleilactone (Majerczyk et al., 2014a).

In this study, the effect of *Sso*Pox V82I, an improved monovariant of *Sso*Pox with enhanced lactonase activity (Billot et al., 2022), was selected and evaluated on *B. thailandensis*. The impact of *Sso*Pox V82I lactonase was assessed through a proteomic approach complemented by phenotypic analysis. The results obtained show that *Sso*Pox V82I treatment significantly disturbs *B. thailandensis* proteome and QS-related phenotypes, including the modulation of antimicrobial compound production.

## 2 Materials and methods

### 2.1 Strains and culture conditions

*Burkholderia thailandensis* E264 (ATCC 700388), Staphylococcus *aureus* clinical isolate, *Chromobacterium violaceum* ATCC 12472 were used in this study. All bacteria were grown at 37°C in Lysogeny Broth (LB) (10 g.L^-1^ NaCl, 10 g.L^-1^ tryptone, 5 g.L^-1^ yeast extract, pH 7), except for *B. thailandensis* which was cultivated in standard succinate media (Meyer, 1978) (6 g.L^-1^ K_2_HPO_4_, 3 g.L^-1^ KH_2_PO_4_, 0.2 g.L^-1^ MgSO_4_.7H_2_O, 1 g.L^-1^ [NH_4_]_2_SO_4_, 4 g.L^-1^ succinic acid, pH adjusted at 7 with NaOH). *B. thailandensis* was inoculated from a single colony and pre-cultivated for 6 hours at 37°C in LB. The pre-culture was diluted 1/1,000 in SSM and 3 mL were added to a 12 well plate. Cultures were incubated for 16 hours with shaking (350 rpm). SsoPox enzyme was added at a final concentration of 0.5 mg.mL^-1^ in the culture. *Saccharomyces cerevisiae* DSM 1333 was cultivated in Yeast extract-Peptone-Dextrose (YPD) medium (Mion et al., 2021) (10 g.L^-1^ yeast extract, 20 g.L^-1^ peptone, 10 g.L^-1^ glucose, pH 6) at 30°C and *Fusarium graminearum* DSM 1096, *Aspergillus niger* DSM 2182 were cultivated in Potato-Dextrose broth (PDB, pH 5) (Sigma Aldrich, USA) at 25°C, respectively.

### 2.2 Production and purification of quorum quenching enzyme

In this study, *Sso*Pox V82I was used as lactonase enzyme. Enzyme production was performed, as described previously (Hiblot et al., 2013; Hraiech et al., 2014), using *Escherichia coli* BL21 (DE3)-pGro7/GroEL strain containing a *Sso*Pox V82I plasmid. Precultures were incubated at 37°C for 16 hours in LB supplemented with ampicillin (100 mg.mL^-1^) and chloramphenicol (34 mg.mL^-1^). Bacteria were then inoculated at 1/100 in ZYP-5052 autoinducer medium supplemented with ampicillin and chloramphenicol, and incubated at 37°C while being stirred (180 rpm). When cultures reached an OD600 nm between 0.8 and 1, bacteria were induced by adding 0.2% L-arabinose for chaperon production and CoCl_2_ at 0.2 mM final, to stabilize the active site of *Sso*Pox V82I enzyme, the temperature was also decreased to 23°C and cultures were grown for 20 hours at 180 rpm. Cells were harvested by centrifugation at 4,000 x g for 20 minutes at 10°C. Pellets were solubilized in lysis buffer (HEPES 50 mM pH 8, NaCl 150 mM, DNAseI 10 µg.mL^-1^, lysozyme 0.25 mg.mL^-1^ and PMSF 0.1 mM). Cells in the lysis buffer were stored at -80°C during at least 16 hours. Thawed cells were sonicated three times for 30 seconds, with an amplitude of 45% (Q700 sonicator^®^, QSonica, USA). Finally, cells were heated at 80°C for 30 minutes and then centrifuged to pellet the debris. The supernatant was collected and saturated with 75% of ammonium sulfate at 4°C for 16 hours to precipitate *Sso*Pox V82I. The enzyme was pelleted down by centrifugation at 10,000 x g for 15 minutes at 10°C. The pellet was resuspended in 8 mL of *Sso*Pox buffer (HEPES 50 mM, NaCl 150 mM and pH 8) and then filtered at 0.8 µm. Ammonium sulfate was removed via desalting (HiPrep 26/10 desalting, ÄKTA pure, GE Healthcare, USA). The sample was concentrated using 30 kDa centricon and then injected into a size-exclusion chromatography column (GF Hiload 16/600 Superdex 75pg) to purify the enzyme. Enzyme purity was then checked by SDS-PAGE and the concentration was measured using a Bradford assay.

### 2.3 Lactonase kinetic parameters

Lactonase kinetic parameters was identified for four *Sso*Pox enzymes: *Sso*Pox W263F, *Sso*Pox W263I, *Sso*Pox V82I-A275G, and *Sso*Pox V82I. The hydrolysis of lactones (C_8_-HSL, 3-OH-C_8_-HSL and 3-OH-C_10_-HSL) was performed in a lactonase buffer (2.5 mM Bicine pH 8.3, 150 mM NaCl, 0.2 mM CoCl_2_, 0.25 mM Cresol purple and 0.5% DMSO, pH adjusted at 8.3 with NaOH), in a range of concentrations between 0 and 2 mM. The reaction of AHL degradation was followed at 577 nm (SynergyHT, BioTek, USA). Mean values were fitted to the Michaelis-Menten equation using Graph-Pad Prism 7.04 software to obtain the catalytic parameters.

### 2.4 AHLs detection

To identify AHLs production in the liquid medium, a *B. thailandensis* 20 mL culture, both untreated and treated with lactonase, was grown for 16 hours. Supernatants were collected by a centrifugation for 10 minutes at 10,000 × g, then 0.22 µm filtered and freeze-dried for 48 hours. The sample was solubilized in 2 mL of ultrapure water. AHLs extraction was performed by adding the same volume of ethyl acetate (v/v) and energetically vortexing the sample. The organic phase was transferred to a glass tube and dried with a nitrogen evaporator. Finally, the dried sample was resuspended in 20 µL in DMSO to concentrate the sample 1,000 times (Rémy et al., 2020). The presence of AHLs in the extract was investigated using the reporter strain *E. coli* MT102. This strain expresses *gfp* in response to AHLs detection (Andersen et al., 2001). The reporter strain was pre-cultivated overnight in LB supplemented with tetracycline 10 µg.mL^-1^ at 30°C, then diluted to 1/10 in fresh LB. A volume of 195 µL of culture was distributed in a 96 well-plate and supplemented with 5 µL of the *B. thailandensis* supernatant extract. The fluorescence was followed every 20 minutes for 16 hours using a plate reader with an excitation wavelength of 485 nm and emission detection at 528 nm.

### 2.5 Biofilm formation

Biofilm formation was measured by crystal violet (Sigma^®^) staining (Hoffman et al., 2005). Planktonic cells were carefully removed from the 12 well plates. Wells were washed with phosphate buffered saline (PBS) and completely dried at 37°C. Then 3 mL of 0.05% crystal violet was added to stain the attached biofilm. Crystal violet was gently removed, and the wells were washed with PBS. The fixed crystal violet was finally dissolved in 3 mL of absolute ethanol, 200 µL were transferred to a 96-well plate, and absorbance was quantified in a microplate reader at 595 nm.

### 2.6 Trypsin proteolysis and tandem mass spectrometry

Cells treated with lactonase or not were harvested, and their proteins were extracted. Four biological replicates were treated for each condition. The protein extracts were diluted with MilliQ water and NuPAGE LDS 3X sample buffer (Invitrogen, USA) supplemented with -mercaptoethanol to obtain a final concentration of proteins of 0.5 µg.µL^-1^ in LDS1X. The samples were heated to 99 ˚C for 5 minutes. For each sample, 20 μL (*i.e.* 10 µg of proteins) were subjected to a short (5 minutes) denaturing electrophoresis on a NuPAGE 4%-12% gradient gel in MES SDS running buffer (50 mM MES ([2-(N-morpholino) ethane sulfonic acid), 50 mM Tris Base, 0.1% SDS, 1 mM EDTA, pH 7.3). After electrophoresis, the gel was briefly stained with SimplyBlue SafeStain (ThermoFisher, USA) and washed extensively with milliQ water. Each proteome was extracted as a single polyacrylamide band. Each sample was processed as previously described (Hartmann et al., 2014) and then proteolyzed with trypsin Gold (Promega, USA) in the presence of ProteaseMax detergent (Promega, USA). A volume of 2 µL of the resulting peptide mixture (50 µL) was injected in a nanoscale C18 PepMap100 capillary column (3 µm, 100 Å, 75 µm id x 50 cm, LC Packings) and resolved with a 90-minutes gradient of acetonitrile (3.2%-20% for 75 minutes followed by 20%-32% for 15 minutes), 0.1% formic acid, at a flow rate of 0.2 µL.min^-1^. The peptides eluting from the column were identified by tandem mass spectrometry with a Q-Exactive HF mass spectrometer (ThermoFisher, USA) operated in data-dependent acquisition mode essentially, as described (Klein et al., 2016). The full scan of peptide ions was acquired at a resolution of 60,000 from *m/z* 350 to 1,500 and with a dynamic exclusion of 10 seconds. Each MS scan was followed by high-energy collisional dissociation and MS/MS scans at a resolution of 15,000 on the 20 most abundant precursor ions, selecting only ions with a charge of 2^+^ or 3^+^. MS/MS spectra were assigned to peptide sequences by the MASCOT Daemon 2.3.2 search engine (Matrix Science) using the *B. thailandensis* ATCC 700388 annotated genome, comprising 5,096 entries and totaling 1,694,428 residues. The search parameters were standard: trypsin as enzyme, a maximum of two possible miss-cleavages, peptide *p*-value below 0.05, oxidation of methionine as variable modification, carbamidomethylation of cysteine as fixed modification, and tolerances of 5 ppm and 0.2 Da for the MS and MS/MS signals, respectively. A protein was considered validated when at least two different peptides were detected (*p*-value below 0.05), resulting in a protein identification false discovery rate below 1%, as verified with a reverse decoy database search. Spectral counts (number of MS/MS spectra assigned per protein) were used as proxy for the abundance of the proteins in each condition. Protein abundances in the two conditions and all biological replicates were compared with the PatternLab Tfold procedure (Carvalho et al., 2012; PMID: 22539673). Each protein sequence with a relative abundance between two conditions of Tfold above 1.5 and *p*-value below 0.05 was selected as differentially detected as previously recommended (Gouveia et al., 2020; PMID: 32133743). The mass spectrometry proteomics data have been deposited to the ProteomeXchange Consortium via the PRIDE partner repository with the dataset identifier PXD040565 and 10.6019/PXD040565.

Treated and untreated condition were analyzed using Partial Least Squares discriminant analysis (PLS-DA) as previously described (Mion et al., 2021). Proteins with a fold change ≥ 1.5 and statistical *p*-value < 0.05 were considered as significantly impacted by the lactonase and were classified using the *Burkholderia* Genome Database (Winsor et al., 2008). The violin plot was constructed using ggplot2 and vioplot packages R software (R-4.1.1) (Wickham, 2016; Adler et al., 2022).

### 2.7 Proteolytic activity

Proteolytic activity was determined by skimmed milk assay (Vial et al., 2008) on LB agar plates (10 g.L^-1^ NaCl, 10 g.L^-1^ tryptone, 5 g.L^-1^ yeast extract, 1.5 g.L^-1^ agar, pH 7) containing 2.8% of skimmed milk powder. After 16 hours of incubation, the supernatant of *B. thailandensis* was collected by centrifugation at 10,000 g for 10 minutes and was filtered through a 0.22 µm filter. Holes were punched in SSM agar plates and a volume of 200 µL of filtered supernatant was poured into. The plates were incubated at room temperature for at least 24 hours. Proteolytic activity was determined by measuring the clear zone surrounding the wells. Each condition was performed in triplicates.

### 2.8 Swarming motility assay

Swarming motility was monitored on 0.7% SSM agar (pH adjusted at 7 with NAOH) by inoculating 3 µL of bacteria into the center of the plate. The SSM agar was supplemented with *Sso*Pox V82I lactonase when indicated, at a final concentration of 0.5 mg.mL^-1^. Plates were incubated for at least 48 hours at 37°C under high humidity. The motility area was measured on ImageJ.

### 2.9 Bactericidal effect of B. thailandensis supernatant against *C. violaceum* and *S. aureus*

The bactericidal effect of *B. thailandensis* supernatants was tested against *C. violaceum* and *S. aureus.* Supernatants from 16 hours cultures of *B. thailandensis* were collected by centrifugation at 10,000 x g for 10 minutes at room temperature and filtered through a 0.22 µm filter. Overnight precultures from *C. violaceum* and *S. aureus* were inoculated at 1/1,000 in fresh LB. A volume of 100 µL of culture was poured into 96-well plates supplemented with 100 µL of *B. thailandensis* supernatant, either untreated or treated with lactonase. The culture was grown at 37°C for 24 hours while being agitated (220 rpm). Cell growth was measured by OD 600 every 30 minutes using a plate reader (Infinite M nano,Tecan, Switzerland). To test whether the bactericidal effect remained without the *B. thailandensis* supernatant, the cells were then serial diluted in PBS and plated on an LB-agar plate. Colony-forming units (CFU) were counted after overnight incubation at 37°C.

### 2.10 Antifungal activity of B. thailandensis towards *F. graminearum* and *A. niger* by microdilution assay

The antifungal activity of *B. thailandensis* was determined by a modified microdilution assay (Piochon et al., 2020). Spores were collected from a Potato-Dextrose Agar (PDA) plate (PDB, 1.5% agar, pH 5) and homogenized by vigorous vortexing in 5 mL sterilized water with 10 µL of tween 80 (Arendrup et al., 2015). The OD 600 nm was then adjusted to 0.09-0.1 corresponding approximately to 10^5^ spores.mL^-1^ (Arendrup et al., 2015). A volume of 750 µL of 10 x serial dilutions (10^-1^,10^-2^,10^-3^,10^-4^) was poured into 24-well plates with 750 µL of *B. thailandensis* filtered supernatants from a 16-hour culture, either untreated or treated with enzyme. Spore formation was observed after 96 hours of incubation at 25°C.

### 2.11 Yeasticidal activity against *S. cerevisiae*

To determine the yeasticidal activity of *B. thailandensis* against *S. cerevisiae*, cultures of *B. thailandensis* either untreated or treated with enzyme, were collected and centrifuged at 10,000 x g for 10 minutes at room temperature. A volume of 750 µL of filtered supernatant was mixed with an equal volume of melted YPD-agar (10 g.L^-1^ yeast extract, 20 g.L^-1^ peptone, 10 g.L^-1^ glucose and 1.5% agar, pH 6). A volume of 10 µL of an overnight culture of *S. cerevisiae* and serial dilutions (10^-2^,10^-3^,10^-4^) was spotted on the dry YPD-agar mixed with supernatant. The plates were incubated for at least 24 hours at 30°C (Mion et al., 2021). In addition, cell-free supernatants from *B. thailandensis* were mixed with an equal volume (100 µL) of serial dilutions (10^-2^, 10^-3^, 10^-4^) from *S. cerevisiae* in 96-well plates to evaluate the yeasticidal effect in liquid culture. Growth was measured at OD 600 nm every 30 minutes for 24 hours at 30°C in a plate reader. Once late stationary phase was reached, cultures were serial-diluted in PBS and 10 µL was spotted on YPD-agar to evaluate the ability of the yeast to recover growth after 24 hours in competition with the supernatant. Agar plates were incubated at 30°C for 24 hours.

### 2.12 Extraction and identification of antimicrobials using liquid chromatography-mass spectrometry (LC-MS)

*B. thailandensis* antimicrobial molecules were extracted as previously described for AHLs. Here, 50 mL of culture medium were freeze-dried and resuspended in 3 mL of water and extracted with an equal volume of ethyl acetate. Organic phases were evaporated under nitrogen flow and resuspended in 50 µL of HPLC-grade methanol. Sterile SSM was used as blank.

Molecules were identified using liquid chromatography coupled with mass spectrometry (LC-MS). For each sample, stored at 4°C, 1 µL was injected into a reverse phase column (Acquity BEH C18 1.7 µm 2.1 × 50 mm, Waters, USA) maintained at 35°C. Compounds were eluted at 0.5 mL.min^-1^ using water and methanol (LC/MS-grade) supplemented with 0.1% of formic acid, with a gradient from 30% to 95% of methanol for 2minutes, 95% of methanol for one minute, and back to the initial composition for 1 minute. Compounds were ionized in the positive mode using a Zspray electrospray ion source: capillary/cone 1.5 kV/20 V, source/desolvation 120°C/250°C. Ions were then monitored using a High Definition MS(E) data independent acquisition method, and the following settings were executed: travelling wave ion mobility survey, 50 *m/z*–1000 *m/z*, 0.1 second scan time, 6 eV low energy ion transfer, and 20–40 eV high energy for collision-induced dissociation of all ions (low/high energy alternate scans). A lockmass correction was applied to adjust mass calibration within each run (Leucin Enkephalin 556.2766 *m/z*). Beforehand, the Vion instrument was calibrated using a Major Mix solution (Waters, USA) to calculate collision cross section (CCS) and mass-to-charge ration (*m/z*). 4D peaks, corresponding to a chromatographic retention time, ion mobility drift time and parents/fragments masses, were then collected from raw data using UNIFI software (version 1.9.4, Waters, USA). Chemical structures were targeted with the following parameters: 2 ppm *m/z* tolerance on parent adducts (H^+^), isotopic profile match of chlorine atoms, and 10 mDa *m/z* tolerance on common bactobolin structure fragments, 383.07712, 312.04000, 294.02944 and respective [M+H]^+^ C_14_H_21_Cl_2_N_2_O_6_, C_11_H_16_Cl_2_NO_5_ and C_11_H_14_Cl_2_NO_4_ identified in MoNa-MassBank of North America (https://mona.fiehnlab.ucdavis.edu/spectra/display/CCMSLIB00004679189).

### 2.13 Statistical analysis

For AHLs detection, biofilm formation, proteolytic activity and motility statistical analyses were performed using Student’s *t*-test in GraphPad Prism 7.04. The significance level (α), or the probability of committing a type I error was set at 0.05.

## 3 Results

### 3.1 *Sso*Pox V82I degrades AHL produced by *B. thailandensis* E264 and decreases biofilm formation

*B. thailandensis* was shown to produce three AHLs: C_8_-HSL, 3-OH-C_8_-HSL and 3-OH-C_10_-HSL (Majerczyk et al., 2014b). The ability of previously reported *Sso*Pox variants (Hiblot et al., 2013; Billot et al., 2022) to degrade these lactones was thus evaluated. Kinetic parameters were determined on synthetic AHLs using a colorimetric assay with four *Sso*Pox variants: *Sso*Pox W263F, *Sso*Pox W263I, *Sso*Pox V82I-A275G and *Sso*Pox V82I. *Sso*Pox W263F efficiently degraded both AHLs with the hydroxyl group, 3-OH-C_8_-HSL and 3-OH-C_10_-HSL, with k_cat_/K_M_ values reaching 2.79 × 10^3^ and 3.49 × 10^3^ M^-1^.s^-1^ respectively but had a poor activity on C_8_-HSL with a k_cat_/K_M_ value of 7.39 × 10^1^ M^-1^.s^-1^. *Sso*Pox W263I showed a better activity on C_8_-HSL degradation, with a k_cat_/K_M_ value reaching 5.75 × 10^3^ M^-1^.s^-1^ as compared to *Sso*Pox W263F, but lower k_cat_/K_M_ were measured against the hydroxylated AHL, 1.88 × 10^2^ and 2.10 × 10^2^ M^-1^.s^-1^, for 3-OH-C_8_-HSL and 3-OH-C_10_-HSL respectively. *Sso*Pox V82I-A275G variant showed the same profile of degradation as *Sso*Pox W263I, with an improvement of k_cat_/K_M_ values on 3-OH-C_10_-HSL. *Sso*Pox V82I presented a similar profile to *Sso*Pox W263F, with an improvement in k_cat_/K_M_ values for C_8_-HSL and 3-OH-C_8_-HSL. *Sso*Pox V82I had the best degradation profile on two synthetic AHL produced by *B. thailandensis*, with the highest activity on C_8_-HSL (4.72 × 10^4^ M^-1^.s^-1^), as compared to the three other variants (**Table 1**). Therefore, *Sso*Pox V82I was selected to further investigate the impact of QQ on *B. thailandensis*.

**Table 1:** Kinetic parameters of different *Sso*Pox variants on *B. thailandensis* AHL.

Enzyme variant	AHLs	k_cat_ (s^-1^)	K_M_ (µM)	k_cat_. $K_{M}^{-1}$ (M^-1^.s^-1^)
*Sso*Pox W263F	C_8_-HSL	0.03 ± 0.02	360.70 ± 71.61	(7.39 ± 6.68) × 10^1^
	3-OH-C_8_-HSL	1.56 ± 0.07	560.00 ± 55.59	(2.79± 0.39) × 10^3^
	3-OH-C_10_-HSL*	1.12 ± 0.05	320. 50 ± 43.62	**(3.49 ± 0.64) × 10^3^**
*Sso*Pox W263I	C_8_-HSL	1.03 ± 0.03	179.00 ± 21.94	(5.75 ± 0.90) × 10^3^
	3-OH-C_8_-HSL	0.21 ± 0.01	1135.00 ± 67.59	(1.88 ± 0.17) × 10^2^
	3-OH-C_10_-HSL	0.10 ± 0.01	490.90 ± 48.40	(2.10 ± 0.29) × 10^2^
*Sso*Pox V82I	C_8_-HSL	3.01 ± 0.06	63.63 ± 7.27	**(4.72 ± 0.64) × 10^4^**
	3-OH-C_8_-HSL	0.90 ± 0.001	301.6 ± 14.99	**(2.98 ± 0.15) × 10^3^**
	3-OH-C_10_-HSL	0.06 ± 0.001	43.86 ± 5.02	(1.48 ± 0.20) × 10^3^
*Sso*Pox V82I-A275G	C_8_-HSL	1.74 ± 0.13	1373 ± 176.60	(1.27 ± 0.25) × 10^3^
	3-OH-C_8_-HSL	0.15 ± 0.01	1394 ± 90.29	(1.07 ± 0.11) × 10^2^
	3-OH-C_10_-HSL*	0.12 ± 0.01	224.20 ± 21.02	(5.29 ± 0.64) × 10^2^

Values represent n= 3 replicates except for data annotated * n=2. The best k_cat_/K_M_ values are highlighted in bold font for each AHL.

**Figure 1:**
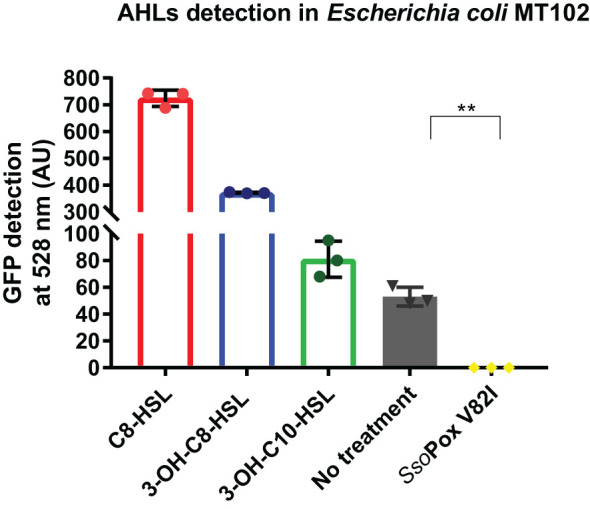
AHLs detection in cell-free extracts of *B. thailandensis*. AHL detection in *B. thailandensis* cell-free extracts using an *E. coli* MT102 reporter strain after cultivation without and with *Sso*Pox V82I (0.5 mg.mL^-1^). C_8_-HSL, 3-OH-C_8_-HSL and 3-OH-C_10_-HSL were used as a positive control at 100 µM. Error bars represent standard deviations for n=3 biological replicates. **p-value < 0.01 according to Student’s *t*-test.

To confirm *Sso*Pox V82I activity on natural AHLs produced by *B. thailandensis* in laboratory conditions, supernatants of *B. thailandensis* were collected after 16 hours of growth in standard succinate medium (SSM) and AHLs were extracted. The detection of AHLs in cell-free extracts was performed using the reporter strain *E. coli* MT102, which produces a fluorescence signal upon the perception of AHLs (Andersen et al., 2001). The fluorescence signal induced by the control with synthetic lactones, at 100 µM, confirmed the detection of the three synthetic AHLs. Furthermore, fluorescence signals were detected for cell-free extracts confirming AHLs production by *B. thailandensis*. AHLs degradation by the QQE, *Sso*Pox V82I, was then confirmed, as the enzymatic treatment completely abolished the detection of AHLs by the reporter strain (**Figure 1**).

**Figure 2:**
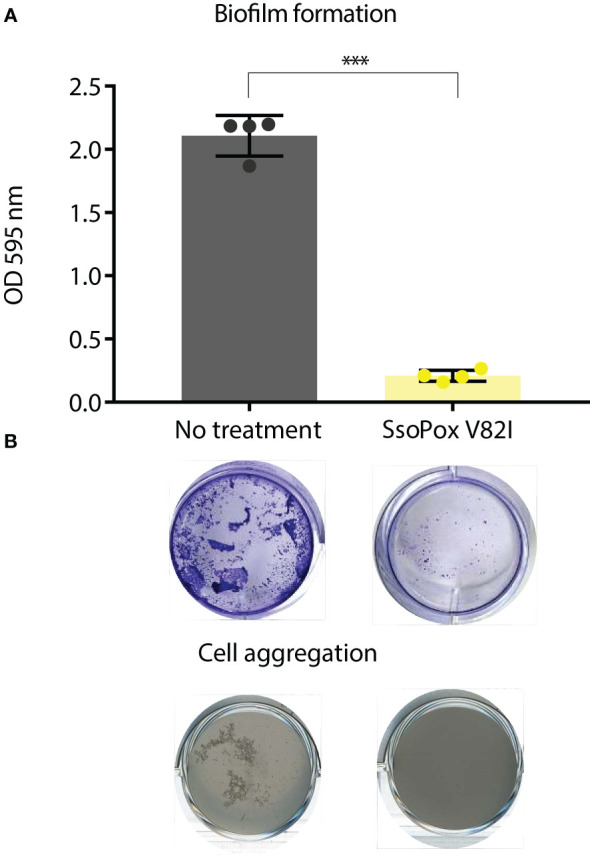
*Sso*Pox V82I disrupts biofilm formation and aggregation in *B. thailandensis*. **(A)** Mean levels of biofilm formation by *B. thailandensis* without enzymatic treatment (gray) and treated with 0.5 mg.mL^-1^ *Sso*Pox V82I (yellow). Error bars represent the standard deviations of n=4 biological replicates. ***p-value < 0.001 according to Student’s *t*-test. Representative pictures of crystal violet staining of biofilm formation are presented below each condition. **(B)** Cell aggregation in shaken culture treated with *Sso*Pox V82I (0.5 mg.mL^-1^) or untreated after 16 hours culture at 37°C.

Finally, as biofilm formation and self-aggregation are known to be under QS regulation in *B. thailandensis*, these phenotypes were used to validate QQ by *Sso*Pox V82I (Chandler et al., 2009; Tseng et al., 2016). Using crystal violet staining, a strong decrease in biofilm formation was measured when *B. thailandensis* was grown in the presence of *Sso*Pox V82I at 0.5 mg.mL^-1^, reducing biofilm formation by more than 90% (**Figure 2A**). In addition, self-aggregation did not occur in cultures treated with *Sso*Pox V82I lactonase, whereas in the untreated condition, aggregation was observed (**Figure 2B**). Taken as a whole, these experimental conditions were chosen to study QQ in *B. thailandensis*. To this end, a global approach combining proteomic and phenotypic analyses was implemented.

### 3.2 Lactonase treatment largely modulates the *B. thailandensis* proteome

**Figure 3:**
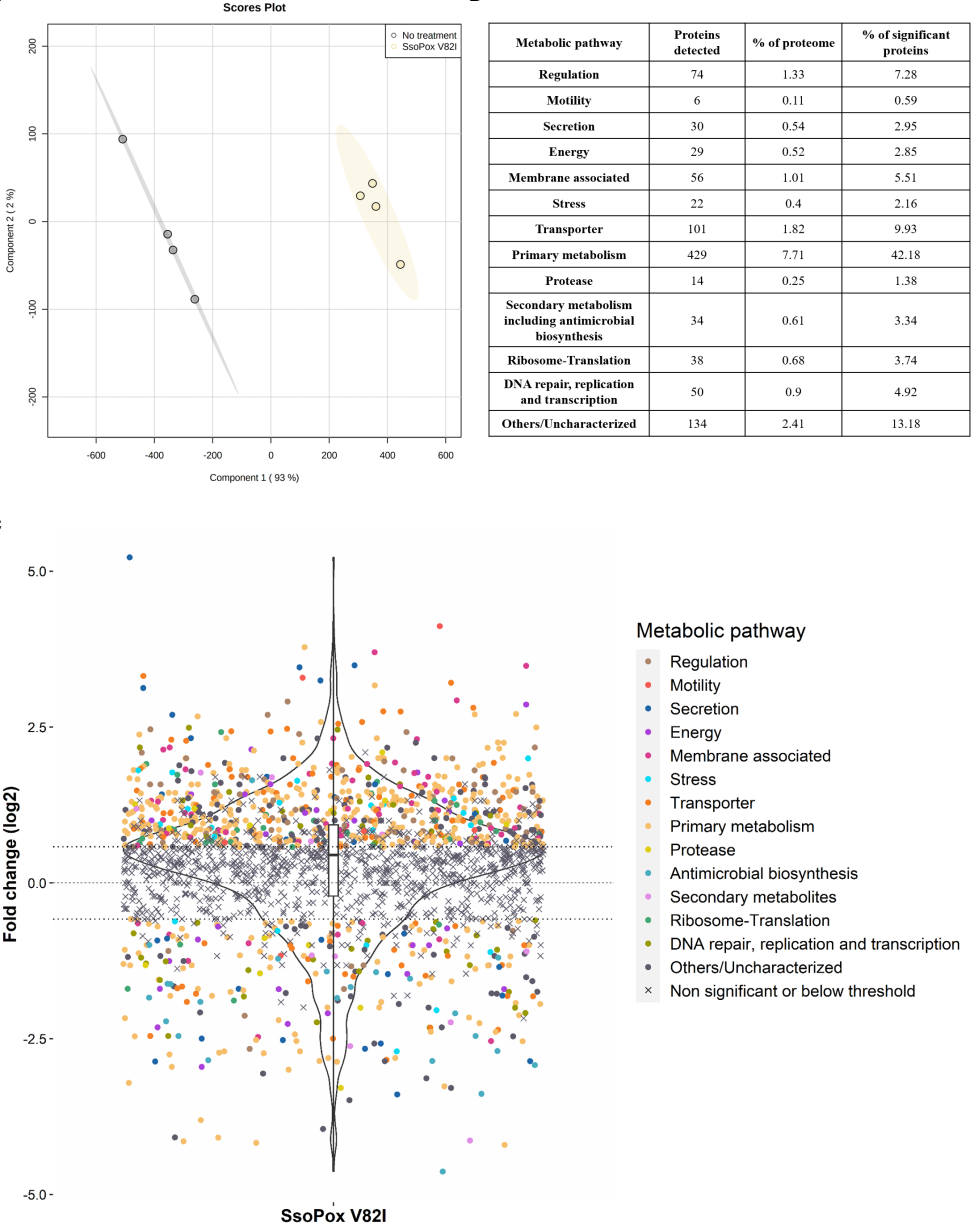
*B. thailandensis* E264 proteome changes upon *Sso*Pox V82I treatment. **(A)** PLS-DA analysis of proteomic analysis of n=4 biological replicates. Untreated samples are represented in gray and samples treated with *Sso*Pox V82I (0.5 mg.mL^-1^) are in yellow. **(B)** Table summarizing the number of proteins impacted by *Sso*Pox V82I treatment and their respective proportion to the *B. thailandensis* proteome or number of significantly impacted proteins. **(C)** Representation of proteomic fold changes as a violin plot. Logarithmic fold changes of 2,495 proteins are plotted and proteins are classified according to their functions. The central line represents a null fold change, meaning no modification occurs with *Sso*Pox V82I treatment. The x=0.58 and x=-0.58 lines correspond respectively to a 50% increase or decrease.

In order to get a complete overview of changes involved by QQ with *Sso*Pox V82I on *B. thailandensis*, proteomic analysis was undertaken. The complete proteome of *B. thailandensis* comprises 5,562 proteins (Proteome ID UP000001930). In this study, 2,495 proteins (i.e. 44.9%) were detected and identified (**Supplementary Data 1**). Using a fold change of ≥ 1.5 and statistical *p*-value < 0.05 as selection criteria, 1,017 proteins (i.e. 18.3% of the entire proteome and 40.8% of the detected proteome) were identified as significantly impacted by *Sso*Pox V82I. Statistical PLS-DA analysis revealed that the enzymatic treatment is the main separation criterion between the two conditions and accounts for 93% of the total variance (**Figure 3A**) indicating the huge impact that QQ and QS have upon *B. thailandensis* proteome.

Clustering by biological function based on the *Burkholderia* Genome Database (Winsor et al., 2008) was applied to significant proteins and 13 groups were identified (**Figure 3B**). Among the significantly impacted proteins, 72% were upregulated, while only 28% were downregulated, indicating that treatment with *Sso*Pox V82I and QQ led to wide changes in protein regulation and activated numerous pathways (**Figure 3C**). Proteins involved in primary metabolism pathways including amino acid biosynthesis, fatty acid metabolism, sugar metabolism, purine metabolism and energy metabolism, were upregulated for over half of these. Several proteins associated with secondary metabolite pathways, including antimicrobial biosynthesis, were identified and were mostly downregulated, except for six proteins. QQ treatment also impacted proteins involved in secretion, transporter and membrane-associated proteins, with 50%, 73% and 84% of upregulated proteins, respectively. Proteins involved in motility and protease were observed and were mainly upregulated (84% and 57%, respectively). Proteins involved in translation, ribosome formation, DNA repair, replication, transcription and regulation were also globally upregulated (**Figures 3B, C**).

Interestingly, among the proteins related to the QS systems, only the BtaR3 protein (WP_009896076.1) was identified in the proteomic dataset and was downregulated (fold change of -2.53) by *Sso*Pox V82I treatment. BtaR3 is related to QS-3 and responsible for 3-OH-C_8_-HSL production. Previous studies have suggested that QS-1 induces QS-3 (Majerczyk et al., 2014a). In addition, several proteins which were detected were previously described as being under QS regulation, such as proteins detected in the secondary metabolites class (**Supplementary Data 1**). In addition, two proteins identified as belonging to secretion system and membrane-associated classes being altered upon lactonase treatment, namely WP_009893771.1 (-7.29 fold change) and WP_009889485.1 (1.64 fold change), were also described as potentially being involved in biofilm formation (Mao et al., 2017).

### 3.3 QQ altered motility and proteolytic activity in *B. thailandensis*

*Sso*Pox V82I lactonase treatment led to several shifts in the *B. thailandensis* proteome. Proteomic analyses identified proteins involved in protease production and motility as being altered upon lactonase treatment (**Figure 3C**).

**Figure 4:**
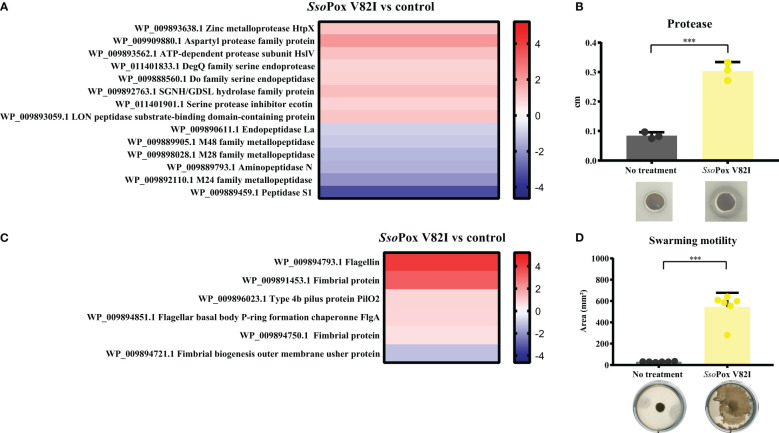
QS disruption in *B. thailandensis* increases proteolytic activity and motility. **(A)** Heatmap representing the fold changes of protease proteins between untreated conditions and those treated with *Sso*Pox V82I (0.5 mg.mL^-1^). **(B)** Mean levels of *B. thailandensis* proteolytic activity on skimmed milk agar plates as measured with ImageJ software of treated *Sso*Pox V82I (0.5 mg.mL^-1^) and untreated conditions. Error bars represent the standard deviations of n=3 biological replicates. ***p-value < 0.001 according to Student’s *t*-test. Pictures of proteolytic halos are presented below each condition. **(C)** Heatmap representing the fold changes of proteins involved in motility between untreated and treated *Sso*Pox V82I (0.5 mg.mL^-1^) conditions. **(D)** Modulation of swarming motility in *B. thailandensis* after lactonase treatment with *Sso*Pox V82I (0.5 mg.mL^-1^) on 0.7% agar plates. Error bars represent the standard deviations of n=6 biological replicates. ***p-value <0.001 according to Student’s *t*-test.

Regarding the protease class, 13 proteins belonged to protease enzymes and one protein was described as a protease inhibitor (WP_011401901.1). Eight of these proteins were upregulated with *Sso*Pox V82I treatment (**Figure 4A**). Most proteases altered by lactonase treatment belonged to the metalloprotease, aspartic protease, or serine protease classification. Two proteins were identified as belonging to the M48 family peptidase (WP_009893638.1 and WP_009889905.1), and proteins in this family are described as HtpX homologs, proteins able to degrade casein(Yoshitani et al., 2019). To support the proteomic results, *in vitro* assays were performed using *B. thailandensis* cell-free supernatants and skimmed milk agar to assess protease activity by casein degradation. Supernatants from untreated cultures showed a weak ability to degrade casein. In comparison, supernatants obtained from lactonase treated cultures harbored an increased capacity to degrade casein, with a degradation halo which was 70% larger (**Figure 4B**).

In addition, proteomic analysis identified six proteins related to motility (**Figure 4C**). One of these proteins was downregulated while five were upregulated. Of them, two proteins were involved in flagella structure: flagellin (WP_009894793.1) a structural protein of the flagellum that plays a role in bacterial surface adhesion (Chaban et al., 2015) and a protein related to the flagella basal body (WP_009894851.1). Flagella proteins are commonly related to swimming and swarming motility (Subramanian and Kearns, 2019). Other proteins involved in motility are related to fimbrial or pili protein (WP_009891453.1 or WP_009896023.1, WP_009894750.1 and WP_009894721.1), and are involved in cellular adhesion (Bzdyl et al., 2022) and twitching motility (Ulrich et al., 2004). To investigate a putative increase in *B. thailandensis* motility upon *Sso*Pox V82I treatment, motility assays were performed. Bacteria grown with or without enzymatic treatment were spotted onto a solid surface (0.7% agar) to evaluate swarming motility. Cells from *Sso*Pox V82I treated condition showed a high increase in swarming motility, representing an 18-fold increase of the motility area as compared to bacteria from the untreated condition (**Figure 4D**).

Proteomic changes upon QQ treatment were thereby supported by *in vitro* assays confirming that QS disruption through *Sso*Pox V82I treatment increases proteolytic activity and motility in *B. thailandensis*. This confirms the validity of the large-scale molecular phenotyping carried out in this study.

### 3.4 QQ treatment modulates antimicrobial production in *B. thailandensis*

To compete with other organisms, *B. thailandensis* produces several antimicrobial molecules (Bach et al., 2021), most of them are under QS regulation (e.g. bactobolin (Seyedsayamdost et al., 2010), thailandamide (Majerczyk et al., 2014a; Majerczyk et al., 2014b), 2-akyl-4-quinolone (Majerczyk et al., 2014a; Majerczyk et al., 2014b) as well as a type VI secretion system (T6SS) (Majerczyk et al., 2016; Lennings et al., 2019).

**Figure 5:**
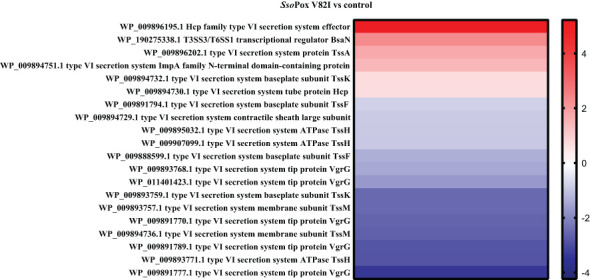
*Sso*Pox V82I treatment impacts T6SS protein levels in *B. thailandensis*. Heatmap of fold-changes in proteins related to T6SS between cells grown in the presence of *Sso*Pox V82I (0.5 mg.mL^-1^) and untreated cells.

**Figure 6:**
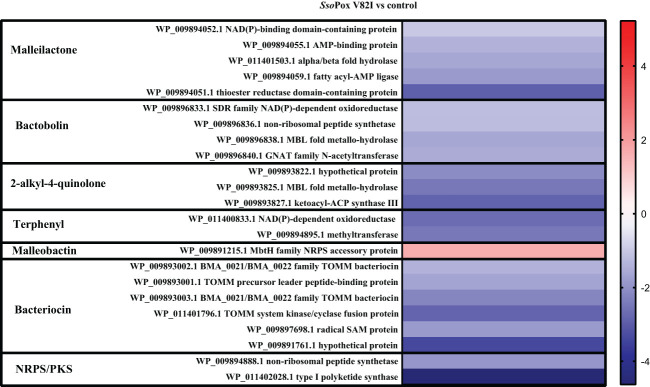
Impact of enzymatic treatment on antimicrobial production. Heatmap of fold-changes of proteins involved in antimicrobial molecule production between cells grown in the presence of *Sso*Pox V82I (0.5 mg.mL^-1^) and untreated cells.

Here, 20 proteins related to T6SS were identified as impacted by the lactonase treatment in the proteomic analyses, most of them being downregulated (i.e. 16 proteins) (**Figure 5**). Similarly, 26 proteins related to the production of bacteriocin, malleilactone, bactobolin, 2-alkyl-4-quinolone, terphenyl and malleobactin were impacted by *Sso*Pox V82I treatment. All the proteins were downregulated except the one involved in malleobactin production (**Figure 6**).

**Figure 7:**
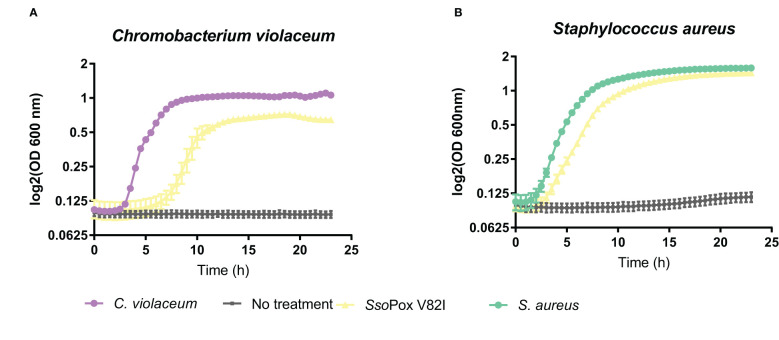
Decrease of bactericidal effect of *B. thailandensis* cell-free supernatants upon QQ. Twenty four hours of bacterial growth kinetics measured by OD 600 nm of **(A)** *C. violaceum* and **(B)** *S. aureus* in the presence of 50% of *B. thailandensis* supernatants obtained from cultures untreated or treated with *Sso*Pox V82I (0.5 mg.mL^-1^). Error bars represent the standard deviations of n= 4 biological replicates for control growth curves without supernatant (in violet and green) and n=6 biological replicates for conditions supplemented with supernatant (in yellow and gray).

To investigate the impact of lactonase treatment on antimicrobial production and its consequences on *B. thailandensis* fitness, different competition assays were designed. A bacterial competition assay was set up using *B. thailandensis* cell-free supernatants, obtained from treated and non-treated cultures, and the Gram-negative bacterium *C. violaceum.* The supernatant obtained from the untreated cultures completely inhibited *C. violaceum* growth. Conversely, when the supernatant was obtained from cultures treated with *Sso*Pox V82I, *C. violaceum* was able to grow (**Figure 7A**). The same experiment was performed with the Gram-positive bacteria *S. aureus* and similarly, *S. aureus* growth was inhibited in the presence of untreated cell-free supernatants, while cell-free supernatants obtained from cultures treated with *Sso*Pox V82I enzyme allowed *S. aureus* to reach the same growth level as bacteria without supernatant (**Figure 7B**). Untreated supernatant inhibited the growth of both bacteria. To determine whether the effect was bacteriostatic or bactericidal, cells were further serially diluted without the addition of supernatant and bacterial survival was evaluated. The bactericidal effect of the supernatant treatment was confirmed with a stronger effect against *C. violaceum* (**Figure S1**). In conclusion, QQ decreased the production of one or several molecules involved in bacterial competition in *B. thailandensis.*

**Figure 8:**
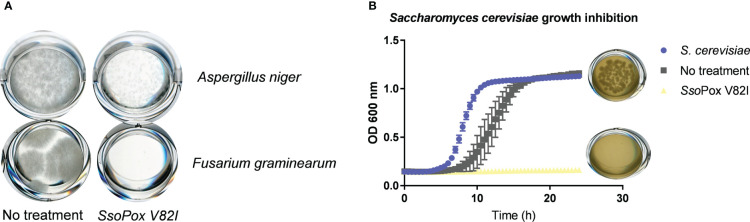
Lactonase treatment increases the antifungal effect of *B. thailandensis* supernatants **(A)** Representative pictures of fungal growth in the presence of *B. thailandensis* cell-free supernatants obtained from cultures treated with *Sso*Pox V82I (0.5 mg.mL^-1^) or not on *A. niger* and *F. graminearum.* Pictures taken after 96 hours of growth at 25°C. **(B)** Effect of *B. thailandensis* cell-free supernatants obtained from cultures treated with *Sso*Pox V82I (0.5 mg.mL^-1^) or untreated against *S. cerevisiae.* Growth was followed by measuring OD at 600 nm for 24 hours. Error bars represent the standard deviations of n= 6 biological replicates. Pictures of *S. cerevisiae* colonies recovered from liquid cultivation are presented next to each condition.

To further characterize the involvement of QS and QQ in antimicrobial compound production, other competition models with eukaryotes were developed. Competition assays were designed using two different fungi: *A. niger* and *F. graminearum.* Cell-free supernatants obtained from *B. thailandensis* cultures untreated or treated with *Sso*Pox V82I were tested on both fungi, and growth was evaluated. In the presence of the untreated supernatant, both fungi were able to grow, while in the presence of the supernatant obtained from treated cultures, growth was drastically reduced or even abolished (**Figure 8A**), indicating that QQ increases the production of antifungal molecules. To complete these observations, the competition assay was applied to the model yeast *S. cerevisiae*. In the same way, colony formation was evaluated in the presence of cell-free supernatants obtained from treated and untreated cultures. *S. cerevisiae* was able to grow in presence of the untreated supernatant while supernatants obtained from *Sso*Pox V82I treated cultures showed a yeasticidal activity against *S. cerevisiae* (**Figure 8B**). These results were confirmed in a microdilution broth assay in which the growth of *S. cerevisiae* was followed over time by measuring absorbance. *S. cerevisiae* growth was completely inhibited with the treated supernatant but recovered a normal growth in the presence of the untreated supernatant (**Figure 8B**). Moreover, this yeasticidal effect was long-lasting, and persisted for weeks. The cultures were also spotted without the addition of supernatant to evaluate any remaining effect of the supernatant. No colony formation was observed in conditions involving the pre-treated supernatant with *Sso*Pox V82I, confirming the increase of yeasticidal activity after QQE treatment (**Figure S2**).

Taken together, these results show that QS modulates the production of antimicrobial compounds in *B. thailandensis*, repressing the expression of a bioactive compound on fungi but inducing the production of another against bacteria.

### 3.5 Bactobolin identification in LC/MS analysis

To further understand the antimicrobial effect detected in the supernatant, ethyl acetate extractions were performed to identify molecules impacted by QQ treatment.

**Figure 9:**
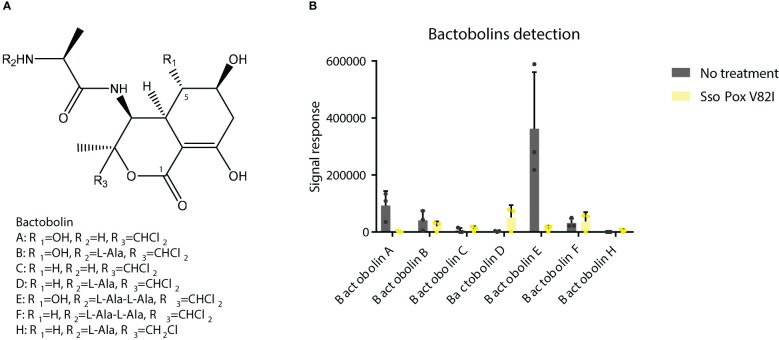
*Sso*Pox treatment decreases the abundance of bactobolins in bacterial supernatants. **(A)** Structures of bactobolins detected in this study. **(B)** Signal detection in LC-MS analysis of several bactobolin molecules after extraction with ethyl acetate of *B. thailandensis* supernatants either untreated or treated with *Sso*Pox V82I (0.5 mg.mL^-1^).

As previously described in this study, the antibiotic activity of the supernatant was shown against *S. aureus* and *C. violaceum* and was significantly inhibited upon lactonase treatment. Proteomic analysis further revealed a significant reduction in proteins involved in antimicrobial molecule production, such as proteins involved in bactobolin biosynthesis. LC/MS analysis were then performed on *B. thailandensis* supernatant to identify the impact of QQ treatment on bactobolin molecules. The antibiotic activity of bactobolin was previously shown against Gram-positive and negative bacteria (Seyedsayamdost et al., 2010; Carr et al., 2011). Eight different bactobolins were reported in *B. thailandensis* (bactobolin A to H) varying in chlorine number (1 or 2), hydroxylation and alanine residues (**Figure 9A**) (Carr et al., 2011). Here, seven bactobolins were detected in the supernatant and were identified using *m/z*, observed retention time, common fragments (identified in bactobolin B spectrum and accessible in MoNA-MassBank of North America), and chlorine atoms. Without lactonase treatment, bactobolin E was the most abundant compound, followed by bactobolin A, bactobolin B and bactobolin F, while the abundance of remaining bactobolins was marginal. Upon lactonase treatment, a significant decrease in bactobolin E and bactobolin A was observed, with a 30- and 50-fold decrease, respectively. The signal response for bactobolin B was also half that of the treated condition. Conversely, bactobolin H was only observed in untreated samples, together with an increase in bactobolin D, but their signal response remained low as compared to bactobolins A and E (**Figure 9B**). Bactobolins impacted by *Sso*Pox V82I treatments were bactobolins with a hydroxylation on C5 (**Figure 9A**). In addition, the protein involved in the hydroxylation reaction (*i.e.* BtaU) was found to be downregulated in proteomic analysis, but was not included among the proteins significantly impacted due to a fold change below the 1.5 threshold (-1.35 fold change decrease). Regarding the three bactobolins with the highest signal response, bactobolin A appears to be the most potent bactobolin, as described previously (Seyedsayamdost et al., 2010; Carr et al., 2011; Chandler et al., 2012b). A previous study also demonstrated that mutations in BtaL and in BtaP shut down the production of bactobolin (Carr et al., 2011), and both proteins were detected in proteomic analysis and were downregulated by *Sso*Pox treatment (**Figure 6**).

In addition, other antimicrobial molecules, such as HMAQ and its derivatives were investigated in sample extracts, as proteins involved in their synthesis were found to be altered in proteomic analysis. *Sso*Pox V82I seemed to increase the signal detection of HAQ C_9_ 2’, HAQNO C_10_ 2’ and HMAQ C_9_, while the signal for HAQNO C_8_ 2’, HAQNO C_9_ 2’, HMAQNO C_9_ 2’ seemed to be decreased (**Figure S3**). However, a wide variability was observed in the samples and did not enable clear conclusions to be drawn about the effect of QQ treatment on the abundance of HMAQ and related compounds.

Supernatant extractions enabled the identification of a decrease in bactobolin signals after QQ treatment, in correlation with the proteomic analysis, that could explain the decrease in bactericidal effect against *S. aureus* and *C. violaceum* in the competition model.

## 4 Discussion

*B. thailandensis* E264 is a soil saprophyte bacteria and is considered to be a non-pathogenic homolog of *B. pseudomallei*. Both bacteria contain three QS-systems which regulate the expression of numerous genes as well as the production of secondary metabolites. In this study, the effect of *Sso*Pox V82I lactonase was evaluated on *B. thailandensis* through proteomic and phenotypic analysis.

AHLs used by *B. thailandensis* were described as C_8_-HSL, 3-OH-C_8_-HSL and 3-OH-C_10_-HSL by LC/LC/MS analysis (Duerkop et al., 2009). Here, *in vitro* degradation assays towards these lactones were used to evaluate the performance of four previously described *Sso*Pox variants, and *Sso*Pox V82I was chosen. The QQ ability of this variant was then confirmed on *B. thailandensis* and a significant reduction in biofilm formation and cell aggregation, two QS-related phenotypes of *B. thailandensis*, was achieved (Chandler et al., 2009; Majerczyk et al., 2014a; Tseng et al., 2016). To get a broader picture of global changes involved by QS and QQ, proteomic analyses were performed.

Proteomic analyses provided evidence that QQ by *Sso*Pox V82I treatment generates a huge shift in *B. thailandensis* with 18.3% of the proteome being significantly impacted. Several metabolic pathways were impacted, including primary metabolites such as sugar and amino acid metabolisms. AHLs synthesis is directly related to methionine metabolism, therefore, in addition to AHLs signal disruption, QQ might also generate an improper balance in the primary metabolism, impacting many metabolic pathways. Proteomic analysis revealed that the treatment with *Sso*Pox V82I results in activation of numerous pathways: 72% of impacted proteins were upregulated, suggesting that QS represses several key functions in *B. thailandensis*. In contrast, previous studies, using other *Sso*Pox variants on *P. aeruginosa* and *C. violaceum* did not induce such an upregulation upon lactonase treatment, suggesting that QS equally upregulated or downregulated proteins on these strains (Rémy et al., 2020; Mion et al., 2021). QS regulation is known to be complex in *B. thailandensis*, and could repress or activate AHL-responsive genes depending on the growth stage (Majerczyk et al., 2014a).

To confirm proteomic changes at the phenotypic level, different bioassays were performed which showed that motility and proteolytic activities were increased after *Sso*Pox V82I treatment and that antimicrobial compound production and secretion were modulated. Indeed, competition assays using cell-free supernatants showed that the production of an antifungal molecule was induced by QQ while the production of an antibacterial molecule was repressed. Motility was already known to be negatively regulated by QS in *B. thailandensis* (Ulrich et al., 2004; Chandler et al., 2009; Passos da Silva et al., 2017), and the increased motility observed after QQ treatment confirmed these results. Regarding proteolytic activity, previous studies did not show any effect on protease production in a QS-mutant strain, suggesting that proteolytic activity was not related to QS (Chandler et al., 2009). Under our conditions and using a QQE, protease activity was increased, as observed on milk agar plates, suggesting that proteolytic activity is negatively regulated by QS in *B. thailandensis*.

Biosynthesis of some antimicrobial molecules and secondary metabolites, in *B. thailandensis*, was also previously described to be QS-controlled (Majerczyk et al., 2014a). In our conditions, QQ treatment by *Sso*Pox V82I led to a decrease in bactericidal effect against *C. violaceum* and *S. aureus*. Several proteins involved in antibiotic molecule synthesis (including malleilactone, terphenyl and bactobolin) were identified as being downregulated by *Sso*Pox in proteomic analyses. Malleilactone is a cytotoxin that contributes to virulence in *B. pseudomallei* and *B. thailandensis* (Klaus et al., 2018), and its bactericidal effect against Gram positive bacteria was described (including in *S. aureus* and *B. subtilis)* (Biggins et al., 2012), and bactericidal activity against *B. subtilis* was reported for terphenyl (Biggins et al., 2011). Proteins related to bacteriocin biosynthesis (Russell et al., 2014; Majerczyk et al., 2016; Mao et al., 2017) were identified and downregulated by lactonase treatment in proteomic analysis. In addition, proteins involved in bactobolin production were decreased after *Sso*Pox V82I treatment. Bactobolin is a well described antibiotic compound against different bacteria such as *S. aureus* (Klaus, 2020) and *C. violaceum* (Chandler et al., 2012a). Synthesis of bactobolin is controlled by QS-2 (3-OH-C_10_-HSL, BtaI2-BtaR2) (Seyedsayamdost et al., 2010; Majerczyk et al., 2014a) and has been described as the sole active antibiotic compound remaining in cell-free supernatants after 0.22 µm filtration (Klaus et al., 2020). Furthermore, LC-MS/MS analysis revealed a reduced response in bactobolin in treated samples. It is, therefore, probable that the bactericidal effect observed without *Sso*Pox V82I treatment is due to bactobolin production. Interestingly, while decreasing antibacterial effect, QQ treatment led to a strong increase in antifungal activity against *A. niger, F. graminearum* and *S. cerevisiae.* A similar increase in antifungal activity was also reported in *Burkholderia ambifaria*, a member of the *Burkholderia cepacia complex*, when *hmqG* or *hmqA*, two genes belonging to the *hmqABCDEFG* operon responsible for HMAQ production and under QS regulation (Coulon et al., 2019), were mutated (Vial et al., 2008). This operon was also described in *B. thailandensis* and *B. pseudomallei* (Vial et al., 2008). In addition to an increase in antifungal activity, *hmqG* or *hmqA* mutations led to an intensification in proteolytic activity (Vial et al., 2008). Here, the HmqG protein (WP_009893822.1) was identified in the proteomic analysis as being downregulated by *Sso*Pox V82I treatment, confirming previous results obtained in *B. ambifaria.* However, when extractions were performed and analyzed in LC-MS, HMAQ molecules and derivatives were identified, but a high variability between samples was observed and did not allow to draw conclusion about the impact of QQ on these molecules. Interestingly, these extractions were tested and, surprisingly, the extracted supernatants had lost their antifungal effect, suggesting a potential alteration of the bioactive compounds during the extraction step.

Previous studies have demonstrated the conservation of QS regulons and QS-regulated functions in *B. pseudomallei* and *B. thailandensis* (Majerczyk et al., 2014b) and within *Burkholderia cepacia complex* (Wopperer et al., 2006). In addition, previous studies investigated QS disruption using QS mutants (Ulrich et al., 2004; Chandler et al., 2009; Tseng et al., 2016) and QQ lactonase (Ulrich, 2004; Wopperer et al., 2006) in *Burkholderia* species have demonstrated that QS is involved in carbon metabolism and the regulation of phenotypes such as biofilm formation, motility and proteolytic activity. Here, the global proteomic approach offers an overview of the implication of QS disruption in *B. thailandensis* and could be extended to other *Burkholderia* species.

Proteomic and phenotypic analyses outline the importance of QS disruption in antimicrobial production modulation and its impact on the interbacterial or interkingdom competition model. Recent studies on several microbiota such as human gut microbiota (Coquant et al., 2021) and human oral cavity microbiota (Muras et al., 2020) have detected the presence of AHLs. In addition, their production could be related to inflammatory diseases in gut and dental plaque formation. In environmental isolates, AHLs have been detected in coral and their presence is related to coral bleaching (Zhou et al., 2020). A recent study using an *Sso*Pox variant demonstrated biofilm disruption in a complex sample from soil and revealed that *Sso*Pox impacted not only bacteria with an AHLs QS system but also bacteria not known to sense or produce AHLs (Schwab et al., 2019). Using a lactonase enzyme, another QQ study confirmed the role of QS on oral microbiota and the impact of a QQE on oral biofilm formation (Parga et al., 2023). These studies highlight the impact of QQ in a heterogeneous population through the modulation of antimicrobial compounds such as antibiotics, fungicides, and secretion systems. These molecules can induce dysbiosis and led to pathology genesis. Taken together, these studies open the way for further investigations of the impact of QQ on microbiota samples, to gain a better understanding of these complex microenvironments.

## 5 Conclusion

Using a combination of phenotypic and molecular approaches we demonstrate that lactonase-mediated quorum quenching strongly alters the behavior and the virulence of *B. thailandensis*, a model strain for studying the highly virulent pathogen *B. pseudomallei*. Biofilm formation, motility, proteolytic activity were strongly impacted upon enzymatic treatment. Interestingly we show that antimicrobial activities of *B. thailandensis* against other microorganisms are highly mediated by the lactonase, bactericidal effect towards both Gram positive and negative strains being significantly decreased while fungicidal activity being tremendously enhanced against both yeast and fungi. This work, combining fine and exhaustive microbiological characterizations together with proteomic analyses, will help to understand the role of QS in bacterial virulence and microbial interactions and will contribute to promote the use of quorum quenchers for controlling pathogens and modulate cooperation and competition in complex microbial populations.

## Data availability statement

The mass spectrometry proteomics data have been deposited to the ProteomeXchange Consortium via the PRIDE partner repository with the dataset identifier PXD040565 and 10.6019/PXD040565.

## Author contributions

MG, LP, ÉC, and DD designed the study; MG, LP, JA, and NA performed the experiments; MG, LP, and DD analyzed the data; MG, LP, ÉC, and DD wrote the manuscript. All authors contributed to the article and approved the submitted version.
