# Supplementary material for: Lactonase-mediated inhibition of quorum sensing largely alters phenotypes, proteome, and antimicrobial activities in Burkholderia thailandensis E264
Source: Front Cell Infect Microbiol. 2023 Jun 2;13:1190859. doi: 10.3389/fcimb.2023.1190859 (PMC10272358; doi:10.3389/fcimb.2023.1190859)
Supplement: Supplementary file 2 [file Table_2.docx]

Supplementary Material

**Supplementary Data 1**-Proteins detected by proteomic analysis and impacted by *Sso*Pox V82I treatment, raw data, fold change and functional classification (See excel file: Supplementary Data 1)

**Figure S1** **-** **Bactericide effect of *B. thailandensis* supernatant.** Effect of *B. thailandensis* supernatant on the growth of **A** *C. violaceum* and **B** *S. aureus.* Serial dilutions of bacteria in PBS after 24 hours of culture in presence of 50 % of *B. thailandensis* supernatant with 0.5 mg.mL^-1^ *Sso*Pox V82I or untreated.

**Figure S2 - Yeasticidal effect of *B. thailandensis* supernatant.** Effect of *B. thailandensis* supernatant on the growth of *S. cerevisiae.* Serial dilutions of *S. cerevisiae* in PBS after 24 hours of culture in presence of 50 % of *B. thailandensis* supernatant with 0.5 mg.ml^-1^ *Sso*Pox V82I or untreated.

**

**Figure S3 – Difference in detection signal of HMAQ molecules and derivatives in LC/MS.** Overview of HMAQ molecules and derivatives detected in supernatant extracted with ethyl acetate untreated or treated (*Sso*Pox V82I 0.5 mg.mL^-1^).
